# Integrating Multiple Hierarchical Parameters to Achieve the Self-Compensation of Scale Factor in a Micro-Electromechanical System Gyroscope

**DOI:** 10.3390/mi15111385

**Published:** 2024-11-16

**Authors:** Rui Zhou, Rang Cui, Daren An, Chong Shen, Yu Bai, Huiliang Cao

**Affiliations:** 1 School of Instrument and Electronics, North University of China, Taiyuan 030051, China; zhourui_nuc@163.com (R.Z.); cuirang@outlook.com (R.C.); andaren0503@163.com (D.A.); shenchong@nuc.edu.cn (C.S.); baiyu19981122@126.com (Y.B.); 2 Chongqing Institute of Microelectronics and Microsystems, Beijing Institute of Technology, Chongqing 401332, China

**Keywords:** MEMS gyroscope, scale factor, PLSR, temperature stability, self-compensation

## Abstract

The scale factor of thermal sensitivity serves as a crucial performance metric for micro-electromechanical system (MEMS) gyroscopes, and is commonly employed to assess the temperature stability of inertial sensors. To improve the temperature stability of the scale factor of MEMS gyroscopes, a self-compensation method is proposed. This is achieved by integrating the primary and secondary relevant parameters of the scale factor using the partial least squares regression (PLSR) algorithm. In this paper, a scale factor prediction model is presented. The model indicates that the resonant frequency and demodulation phase angle are the primary correlation terms of the scale factor, while the drive control voltage and quadrature feedback voltage are the secondary correlation terms of the scale factor. By employing a weighted fusion of correlated terms through PLSR, the scale factor for temperature sensitivity is markedly enhanced by leveraging the predicted results to compensate for the output. The results indicate that the maximum error of the predicted scale factor is 0.124% within the temperature range of −40 °C to 60 °C, and the temperature sensitivity of the scale factor decreases from 6180 ppm/°C to 9.39 ppm/°C.

## 1. Introduction

In recent years, with the continuous advancement of technologies in fields such as inertial navigation [1] and attitude control [2], the demands for inertial devices have become increasingly rigorous. Conventional laser and fiber gyroscopes [3] are unsuitable for the majority of applications due to their bulkiness and high cost. This has led to the development of MEMS (micro-electromechanical system) gyroscopes [4]. MEMS gyroscopes offer the advantages of compact size, lightweight design, cost-effectiveness, and scalable production, making them increasingly preferred for engineering applications. However, compared with other types of gyroscopes, MEMS gyroscopes have poor index parameters, such as scale factor (*SF*), and more technical research is needed to solve these problems. Scale factor is a crucial parameter for evaluating the performance of a gyroscope, as it quantifies the proportionality between the angular rates of the output and input. When the ambient temperature fluctuates, it induces changes in the material properties of the gyroscope structure [5], consequently leading to an indirect alteration in the scale factor. This will impact the measurement performance of the gyroscope, thereby failing to meet the requirements for high-precision navigation and attitude control. Hence, investigating temperature compensation methods for scale factor remains pivotal in enhancing the measurement performance of gyroscopes.

The compensation and calibration of the scale factor temperature drift in MEMS gyroscopes remains a prominent area of research. Among the proposed methods, existing compensation approaches are primarily categorized into three groups: mechanical structure correction [6], electrical circuit correction, and signal post-processing [7]. In [8], the authors presented a design for a micro-vibration driving and sensing platform comprising a three-degree-of-freedom micro-motorized stage capable of providing piezoelectric driving and compensating for undesired off-axis motion while offering X/Y tilt reference stimuli. This method enables long-term drift compensation of the scale factor but necessitates advanced processing technology for the structure. For electrical circuit correction, most researchers devise peripheral circuits or ASIC interface circuits to achieve temperature compensation of the scale factor. In [9], Cao et al. utilize thermistors combined with peripheral circuits to adjust the amplitude of the driving mode and gain of the sense loop, ultimately achieving the temperature coefficient correction of the scale factor. Nevertheless, this approach introduces errors into the measurement system due to the temperature sensor, and it is not possible to achieve intelligent calibration using analogue circuits. Additionally, in [10], an application-specific integrated circuit (ASIC) dedicated to MEMS gyroscope interfacing is proposed to enhance gyroscope temperature adaptability through temperature compensation. But this method may not be practically feasible in engineering applications due to its lengthy development cycle, high cost, and limited universality. The most prevalent compensation method continues to rely on signal post-processing to rectify parameters through the utilization of existing gyroscope measurement data. The most straightforward method is to fit the collected data with temperature sensor data in order to subsequently derive a relationship between temperature coefficients before performing output compensation [11]. However, this method’s performance hinges on algorithm model accuracy and lacks universal applicability. In addition, there are techniques available to address this issue, such as the application of virtual Coriolis force [12,13] to the gyroscope, which results in the introduction of coupling into the gyroscope’s sense output.

This paper presents a method for compensating the temperature sensitivity of the gyroscope scale factor. Initially, key terms related to the scale factor, such as resonant frequency and demodulation phase angle, and secondary relevant terms like drive control voltage and quadrature feedback voltage, are identified through a self-compensation system. Subsequently, multi-parameter fusion is employed to forecast the scale factor parameters using the partial least squares regression (PLSR) algorithm [14]. Finally, compensation is applied to the output in order to achieve the temperature drift compensation of the scale factor. The concept of multi-parameter fusion compensation is illustrated in [15], with notable distinctions from previous studies, including the following: (1) Comprehensive consideration of multiple measurement parameters for fusion, encompassing most influencing factors without introducing additional disturbances that may compromise system robustness. (2) Reduced complexity of the compensation system, leading to less resource consumption by simple compensation algorithms, which saves costs while enhancing method applicability. (3) Optimization of the scale factor prediction model, resulting in significant final compensation effects.

The paper is organized as follows: Section 2 presents the operational principle of the S-beam vibration ring gyroscope (SVRG). Section 3 examines the factors contributing to scale factor variation with temperature and delineates the temperature drift characteristics of the scale factor. Section 4 investigates the relationship between various temperature parameters and establishes a predictive model for scale factor. Additionally, it introduces the PLSR algorithm prediction process. Section 5 validates the feasibility of the temperature compensation scheme through experimental verification. Finally, in Section 6, a summary of the paper is provided.

## 2. Description of MEMS S-Beam Vibration Ring Gyroscope

**Figure 1:**
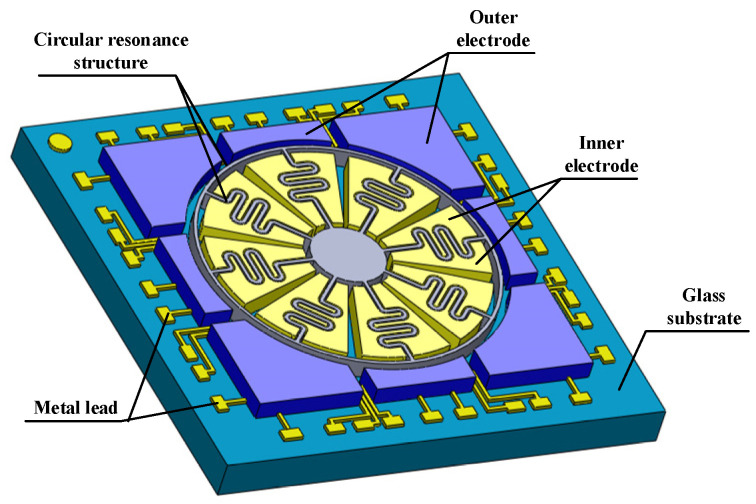
SVRG structure chip diagram.

This paper verifies the effectiveness of the proposed method using an SVRG [16] as an illustrative example. The SVRG features a structure composed of single-crystal silicon and glass. To enhance its overload resistance and capacitive sensor sensitivity, the SVRG resonator utilizes a deep reactive ion etching (DRIE) process with a high aspect ratio [17]. The fabrication process employs a 300 μm thick 4-inch heavily doped single-crystal silicon wafer with 500 μm thick 4-inch Pyrex7740 borosilicate glass. The associated processing comprises four stages: silicon backside etching, electrode patterning on the glass substrate, silicon and glass anode bonding and thinning, and silicon structure release. The 3D structure view of the SVRG is shown as Figure 1.

The gyroscope primarily consists of a ring resonator, 24 control electrodes arranged around the ring resonator, and a glass base structure with graphite metal leads. The central anchor point of the ring resonator is bonded to the glass substrate using an anode bonding method and is connected to the ring mass block through an S-shaped vibration elastic beam. The centerline of each elastic beam forms angles of 0°, 45°, 90°, 135°, 180°, 225°, 270°, and 315° with the *x*-axis, aligning with the intrinsic vibration modes of the ring resonator. This configuration effectively mitigates the impact of environmental damping and stiffness coupling errors on the drive and sense modes, while enhancing the device’s signal-to-noise ratio and angle measurement accuracy.

**Figure 2:**
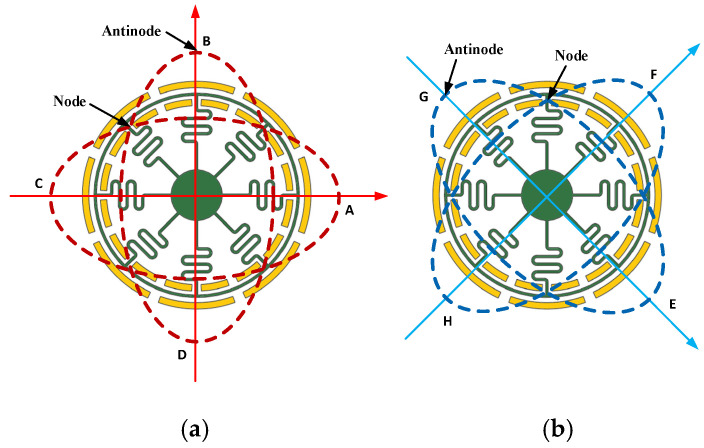
(**a**) Vibrational form of drive mode. (**b**) Vibrational form of sense mode. SVRG’s primary and secondary modal vibration forms.

The sensitive unit of the SVRG is a circular resonant structure that shares a driving and sense mode, with its movement characterized by a deformation vibration mode. The ring-shaped vibratory gyroscope operates akin to the hemispherical resonant gyroscope, generally manifesting as a second-order eigenmode standing wave (wave number n = 2). It exhibits two fundamental working modes, namely the first basic mode and the second basic mode, as depicted in Figure 2. The vibration form of the driving mode (main mode) is illustrated in Figure 2a. Under normal operational conditions, the circular resonator vibrates along a fixed frequency on the red dashed line under a periodic driving force perpendicular to it. The wave nodes are situated at points A (0°), B (90°), C (180°), and D (270°). When an angular velocity Ω_z_ input is applied perpendicular to the forced vibration direction along the *Z* axis, the Coriolis effect causes the bending of the resonator along a blue line in another direction perpendicular to this input. This represents the sense mode (submode). Wave nodes occur at points E (45°), F (135°), G (225°), and H (315°) during vibration. Throughout this process, position wave nodes and crests appear periodically, forming a standing wave, as shown in Figure 2b. When excited by driving voltage alone, only main-mode vibrations are observed. However, when rotating with its base, the Coriolis inertial force induces submode vibrations within this circular resonant structure. The Coriolis energy is interconverted between these two vibration modes.

**Figure 3:**
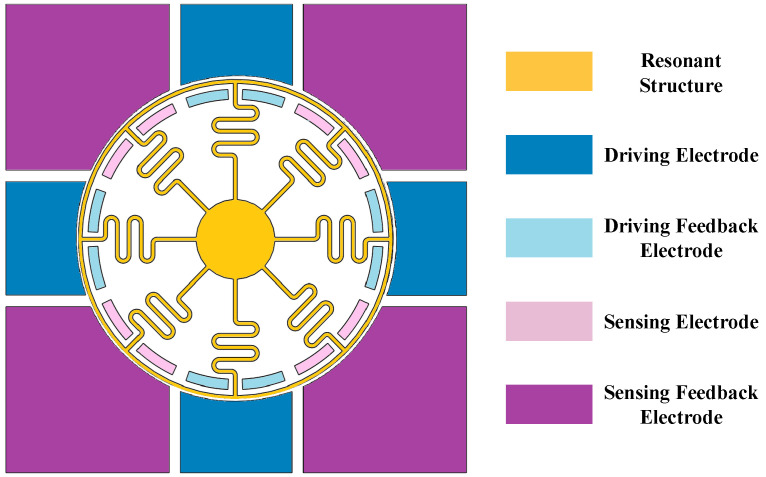
Electrode distribution of SVRG.

This structure employs the principle of electrostatic drive to detect changes in the capacitance of the electrodes and observe angular velocity. The gyroscope is equipped with a total of twenty-four electrodes, which are divided into eight groups of three differential electrodes each, primarily utilized for controlling and tuning the circular resonator. Figure 3 illustrates the electrode allocation of the gyroscope, encompassing driving electrodes, driving feedback electrodes, sensing electrodes, and sensing feedback electrodes. By encapsulating the SVRG chip in a leadless chip carrier (LCC) package and subjecting it to vacuum treatment, the quality factor of the gyroscope can be enhanced, thereby improving its mechanical sensitivity.

## 3. Temperature Sensitivity Analysis

### 3.1. Analysis of Scale Factor and Zero Bias Variation with Temperature

**Figure 4:**
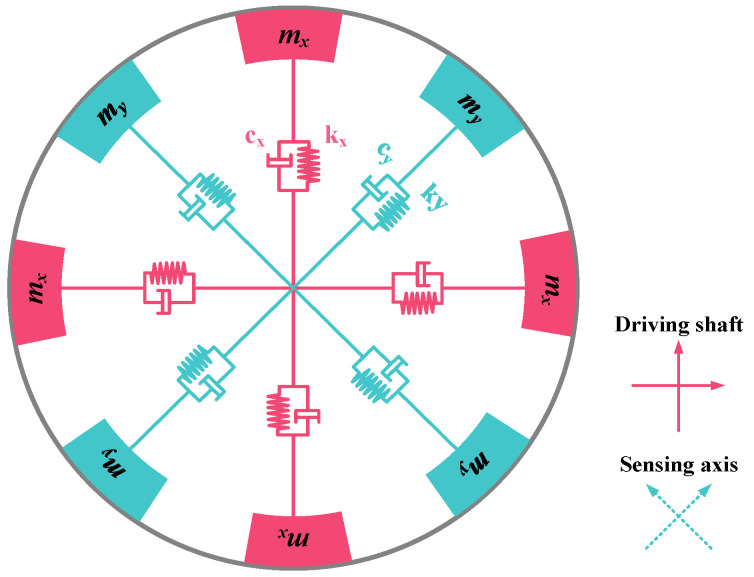
Mechanical model of SVRG.

The SVRG mechanical model can be represented by the equivalent of eight second-order systems, each consisting of a “spring–damper–mass block”, as depicted in Figure 4. Owing to the complete symmetry of the structure, the parameters for both driving and detection beam supports on the resonator are identical in magnitude but differ in orientation, necessitating the analysis of only one pair of second-order systems.

In the process of fabrication, gyroscopes are inevitably affected by manufacturing errors, leading to an imperfect structure. Therefore, when analyzing the mechanical model, it is essential to consider the coupling between stiffness and damping in both the driving axis and sensing axis. The kinematic equations of the non-ideal MEMS gyroscope structure can be expressed as follows:

(1) $$\left\{\begin{array}{l} m_{x}\ddot{x}+b_{x}\dot{x}+k_{x}x+b_{yx}\dot{y}+k_{yx}y=F_{x}+2m_{x}\lambda \dot{y}\Omega_{z}\\ m_{y}\ddot{y}+b_{y}\dot{y}+k_{y}y+b_{xy}\dot{x}+k_{xy}x=F_{y}-2m_{y}\lambda \dot{x}\Omega_{z} \end{array}\right.$$

In this equation, *x* and *y* denote the displacements of the drive and sense axes, while *m_x_* and *m_y_* represent the equivalent masses of these axes. The stiffness coefficients are denoted by *k_x_*, *k_y_*, *k_xy_*, and *k_yx_*, whereas *b_x_*, *b_y_*, *b_xy_*, and *b_yx_* stand for the damping coefficients. Ω*_z_* represents the input angular velocity; *F_x_* and *F_y_* indicate the external excitation forces on the driving and sensing axes, respectively; and *λ* denotes the Coriolis coupling coefficient.

Based on the operating characteristics of the ring solid fluctuation gyroscope and the characteristics of the input signal, the dynamical equations can be approximated as follows: during the operation of the gyroscope, the sensing and driving modes are vibrating at the same frequency, and the vibration displacements of the sensing modes are much smaller than those of the driving modes. *x* >> *y* and $\dot{x}\gg \dot{y}$. Based on the above approximation, (1) can be further simplified as follows:

(2) $$\left\{\begin{array}{l} m_{x}\ddot{x}+b_{x}\dot{x}+k_{x}x=F_{x}\\ m_{y}\ddot{y}+b_{y}\dot{y}+k_{y}y+b_{xy}\dot{x}+k_{xy}x=F_{y}-2m_{y}\lambda \dot{x}\Omega_{z} \end{array}\right.$$

The electrostatic driving force applied to the gyro drive shaft is *F_x_* = *V_d_*sin(*ω_d_t*), *V_d_* is the electrostatic driving force amplitude, and *ω_d_* is the resonant frequency of the driving mode; then, the displacement of the driving mode is

(3) $$\left\{\begin{array}{l} x\left(t\right)=A_{x}\sin\left(\omega_{d}t+\varphi_{x}\right)\\ A_{x}=\frac{V_{d}/m_{x}}{\omega_{x}^{2}\sqrt{{\left(1-\frac{\omega_{d}^{2}}{\omega_{x}^{2}}\right)}^{2}-{\left(2\zeta_{1}\frac{\omega_{d}}{\omega_{x}}\right)}^{2}}}\\ \varphi_{x}=\arctan\left(\frac{2\zeta_{1}\omega_{x}\omega_{d}}{\omega_{x}^{2}-\omega_{d}^{2}}\right) \end{array}\right.$$

where *A_x_* is the drive displacement amplitude; *φ_x_* is the drive phase; *ω_x_* is the drive modal vibration frequency; and the damping ratio ζ_1_ = *b_x_*/2*m_x_ω_x_*. When the gyroscope input axis rotates at Ω*_z_* angular velocity relative to the inertial space, the force in the detection direction of the gyroscope ring resonator sensitive structure is

(4) $$F_{y}=2m_{y}\lambda \Omega_{z}A_{x}\omega_{d}\cos\left(\omega_{d}t+\varphi_{y}\right)+b_{xy}A_{x}\omega_{d}\cos\left(\omega_{d}t+\varphi_{y}\right)+k_{xy}A_{x}\sin\left(\omega_{d}t+\varphi_{y}\right)$$

In open-loop mode, the final voltage output signal is obtained by the multiplicative demodulation of the gyroscope after the action of the Coriolis force as

(5) $$V_{se}=\left(m_{y}\lambda \Omega_{z}+b_{xy}\right)A_{x}\omega_{d}K_{cv}G\cos\left(\varphi_{y}\right)$$

In (5), *K_cv_* is the capacitance–voltage conversion factor, $\varphi_{y}=-arctan\frac{\omega_{y}\omega_{d}}{Q_{y}\left(\omega_{y}^{2}-\omega_{d}^{2}\right)}$ corresponds to the vibrational phase in the direction of the sensed modes, *ω_y_* is the vibrational frequency of the sensed modes, and *G* is the sensed modal transfer coefficient of the gyroscope. Then, the *SF* and bias of the gyroscope output in open-loop mode are calculated as follows:

(6) $$\begin{array}{c} SF=m_{y}\lambda A_{x}\omega_{d}K_{cv}G\cos\left(\varphi_{y}\right)\\ Bias=b_{xy}A_{x}\omega_{d}K_{cv}G\cos\left(\varphi_{y}\right) \end{array}$$

Reference [18] suggests that temperature can influence the mechanical structural size and elastic modulus of the MEMS gyroscope material. While the structural changes have minimal impact on the gyroscope performance and may be disregarded, alterations in the material’s elastic modulus cannot be overlooked, as they will affect the stiffness of the gyroscope structure and lead to variations in its resonance frequency. The relationship between temperature and resonance frequency can be approximated as linear.

(7) $$\omega_{d}\left(T\right)=\omega_{d}\left(T_{0}\right)\left[1+\frac{1}{2}k_{E}\left(T-T_{0}\right)\right]$$

*T*_0_ represents the ambient temperature, i.e., *T*_0_ = 300 K; *k_E_* is the temperature coefficient of the elastic modulus of the silicon material, which ranges from −7.5 × 10^−5^ to −2.5 × 10^−5^. Based on (7), the resonant frequency will decrease in direct proportion to the temperature rise. Derived from the formula, *φ_y_* will also diminish with increasing temperature. The coefficient of capacitance–voltage conversion (*K_cv_*) varies with temperature and can be utilized to elucidate the variations in *SF* and bias, as indicated in (6). Generally, the relationship between *SF*, bias, and temperature for MEMS gyroscopes is non-linear. However, for silicon MEMS, it can be assumed that this change is linear within a specific temperature range.

### 3.2. Temperature Drift Characteristics

The operation of an MEMS gyroscope relies on the energy exchange between two vibration modes: the driving mode, which is continuously excited in its resonant state, and the sensing mode, which measures the angular velocity. The gyroscope’s output signal is typically represented as follows:

(8) $$V_{out}=\left(SF\right)\times \left(\Omega_{z}+B\right)$$

The output electrical signal comprises *SF*, true angular velocity Ω*_z_*, and bias *B*. Bias *B* encompasses a constant bias error *B*_0_ and a temperature drift bias error *B_T_*. *B_T_* is influenced by temperature and can be mitigated through compensation methods to minimize its impact on the gyroscope output.

(9) $$B=B_{0}+B_{T}$$

The impact of temperature on gyroscope systems primarily affects both the control circuit and the external structural parameters of the gyroscope. In the printed circuit board control system, temperature variations cause drifts in resistor and capacitor values, which in turn lead to changes in loop gain and the signal phase within the control system. For the SVRG, which is mainly composed of silicon, temperature fluctuations influence the elastic modulus, dimensions, and stiffness of the gyroscope structure, resulting in variations in resonant frequency and quality factors. Both the temperature error term in *SF* and the deterministic drift in *B* contribute to a noticeable temperature-induced drift in the output, ultimately causing inconsistent measurement results across different thermal environments.

The most prevalent method for temperature compensation is to curve-fit the collected gyroscope output signal and temperature signal in order to establish a model for the temperature error of the gyroscope. This model is then used as an input to an algorithmic process applied to the output signal, thereby achieving the desired compensation effect. This method necessitates a substantial quantity of data as an a priori condition; the experimental operation is intricate and complex, and it is susceptible to the introduction of noise from the temperature sensor into the output signal. In order to address the aforementioned issues, this paper puts forth a novel approach to gyroscope scale factor compensation. The method employs PLSR to predict multiple temperature-varying parameter variables, thereby enabling the prediction of the gyroscope scale factor and the completion of the temperature-sensitive compensation of the scale factor.

## 4. Temperature Compensation Strategy

### 4.1. Temperature Correlation Analysis

**Figure 5:**
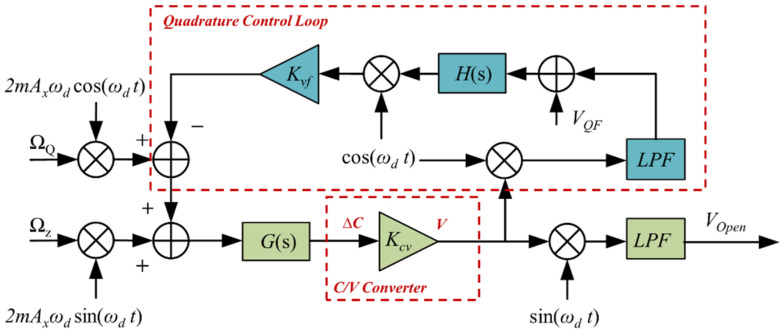
Block diagram of gyroscope’s sense mode of operation.

Figure 5 illustrates the schematic of the gyroscope’s sense mode of operation. It consists of a quadrature control loop and a Coriolis signal output section, where the Coriolis signal output section outputs the gyroscope angular velocity measurement information, while the quadrature control loop is used to reduce the quadrature error [19]. The equivalent input angular rate Ω*_Q_* of the quadrature coupling error can be expressed as follows:

(10) $$\Omega_{Q}=\frac{k_{xy}}{2\omega_{d}m_{y}}$$

Combined with the quadrature signals in (4), a force balance analysis of the quadrature control loop is obtained:

(11) $$V_{QF}=\frac{2m_{y}\Omega_{Q}A_{x}\omega_{d}}{K_{vf}K_{cv}H}$$

*V_QE_* is the quadrature feedback force–voltage magnitude, *K_vf_* is the voltage-to-force conversion factor. *H* is the corrected transfer function. From (11), it can be seen that the temperature will further affect the magnitude of the quadrature feedback force by affecting the resonance frequency, demodulation phase angle, etc., and the quadrature feedback force will also change with temperature.

In order to ensure that the gyroscope accurately detects the input angular rate, we need to ensure that its drive vibration rate is constant. For this purpose, an automatic gain control (AGC) method based on parameter setting is used to realize the closed-loop control of the drive amplitude [20]. The drive control voltage *V_DF_* is also affected by the resonant frequency and can be expressed as follows:

(12) $$V_{DF}=\frac{A_{x}\omega_{d}}{K_{vf}K_{cv}}$$

**Figure 6:**
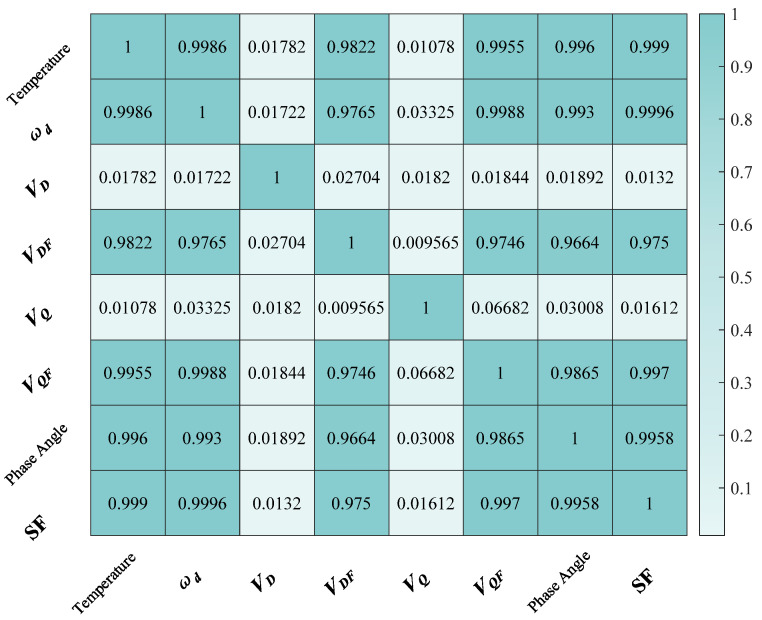
Heatmap of correlation analysis for each parameter.

In the content analysis mentioned in Section 3, the scale factor is affected by multiple variables. Therefore, it is feasible to combine multiple variables affected by temperature to discover the relationship between the scale factor and temperature. Figure 6 demonstrates the relational analysis of each variable parameter with respect to temperature.

The changes in these parameters in the full-temperature environment are essentially linear, and the multivariate fitting of these variables can be used for the prediction of the scale factor. The following model was developed for predicting the scale factor:

(13) $$SF_{pre}=\alpha_{0}+\alpha_{1}\cdot f_{1}\left(\omega_{d}\right)+\alpha_{2}\cdot f_{2}\left(V_{DF}\right)+\alpha_{3}\cdot f_{3}\left(V_{QF}\right)+\alpha_{4}\cdot f_{4}\left(\varphi_{y}\right)$$

*α_i_* is the correlation fitting coefficient and *f_i_*(·) is the fitting function corresponding to a particular variable.

### 4.2. Partial Least Squares Regression Algorithm Parameter Prediction

The partial least squares regression (PLSR) algorithm is an effective multivariate statistical analysis method that combines the features of principal component analysis (PCA) [21] and multiple linear regression (MLR) [22]. Compared with other regression methods, the PLSR algorithm is able to obtain more satisfactory prediction results with a smaller number of samples, and its operation places more emphasis on the relationship between variables than on the number of samples. Therefore, it is appropriate to use PLSR to realize the prediction of the results of the model constructed in (11). The PLSR prediction process is mainly divided into two phases: principal component extraction and iterative regression analysis.

(1) Establishment of the variable system.

It is first necessary to establish the independent variable system ***X*** and the dependent variable system ***Y***. The resonant frequencies, quadrature feedback voltages, and demodulated phase angles in different temperature environments constitute the independent variable system ***X*** = {***ω_d_***, ***V_DF_***, ***V_QF_***, ***φ_y_***}, and the actual measured values of *SF* at different temperatures constitute the dependent variable system ***Y*** = {***SF***}.

(2) Principal component extraction.

After normalizing ***X*** and ***Y*** with zero mean and one variance, the matrices ***X***_0_ and ***Y***_0_ are obtained. Calculate the standard eigenvectors ***w***_1_ and ***c***_1_ for the largest eigenvalues of ***X***_0_***^T^Y***_0_***Y***_0_***^T^X***_0_ and ***Y***_0_***^T^X***_0×0_***^T^Y***_0_.

Principal component *u*_1_ is extracted from the independent variable system ***X*** and principal component *v*_1_ is extracted from the dependent variable system ***Y***. It is guaranteed that *u*_1_ and *v*_1_ extract as much information as possible about the variance in their respective systems and that the correlation between *u*_1_ and *v*_1_ is maximized. The constraints are as follows:

(14) $$\left\{\begin{array}{l} u_{1}=X_{0}w_{1}\\ v_{1}=Y_{0}c_{1}\\ \begin{array}{cc} \max<u_{1},v_{1}> & s.t\left\{\begin{array}{l} {\left\|w_{1}\right\|}^{2}=1\\ {\left\|c_{1}\right\|}^{2}=1 \end{array}\right. \end{array} \end{array}\right.$$

(3) Calculation of regression coefficients.

The regression equation of ***X***_0_ and ***Y***_0_ on *u*_1_ is shown in Equation (15).

(15) $$\begin{array}{l} X_{0}=u_{1}p_{1}^{T}+X_{1}\\ Y_{0}=u_{1}r_{1}^{T}+Y_{1} \end{array}$$

***p***_1_ and ***r***_1_ are regression coefficients:

(16) $$\begin{array}{l} p_{1}=\frac{X_{0}^{T}u_{1}}{{\left\|u_{1}\right\|}^{2}}\\ r_{1}=\frac{Y_{0}^{T}u_{1}}{{\left\|u_{1}\right\|}^{2}} \end{array}$$

(4) Iterative regression analysis.

Repeat operations (2) and (3) with ***X_k_*** and ***Y_k_*** (*k* = 1,…, *A*); *A* equals the rank of matrix ***X***) instead of ***X_k_***_−__1_ and ***Y_k_***_−__1_, respectively, until *k* principal components *u*_1_, *u*_2_, …, *u_k_* are extracted and *k* regression coefficients ***p***_1_, ***p***_2_, …, ***p_k_*** and ***r***_1_, ***r***_2_, …, ***r_k_*** are achieved. The final regression model is as follows:

(17) $$\begin{array}{l} X_{0}=u_{1}p_{1}^{T}+\cdots +u_{k}p_{k}^{T}+X_{k}\\ Y_{0}=u_{1}r_{1}^{T}+\cdots +u_{k}r_{k}^{T}+Y_{k} \end{array}$$

Substituting $u_{1}=w_{1}^{T}X$ into Equation (15) gives the final regression equation, obtained as

(18) $$SF_{pre}=\alpha_{0}+\alpha_{1}\omega_{d}+\alpha_{2}V_{DF}+\alpha_{3}V_{QF}+\alpha_{4}\varphi_{y}$$

**Figure 7:**
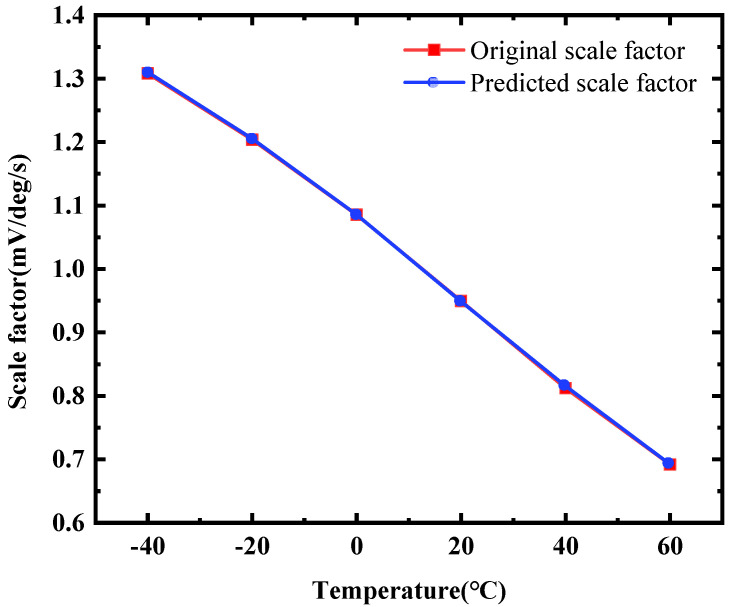
Scale factor prediction results.

Figure 7 shows the comparison of the PLSR predicted scale factor and the actual measured scale factor results; it can be seen that the maximum prediction error is 0.124% and the root mean square error (RMSE) is 0.0018. The results prove that the model predicts well.

The temperature compensation of the scale factor can be realized by using the predicted scale factor to compensate the gyroscope output according to (19). *SF_pre_*(T_0_) denotes the predicted value of the scale factor at the standard temperature, and *SF_pre_*(T) denotes the predicted value of the scale factor at temperature T. *V_ucom_*(T) denotes the uncompensated gyroscope output at temperature T, and *V_com_*(T) denotes the compensated gyroscope output.

## 5. Experimental Validation

### 5.1. Control System Establishment

**Figure 8:**
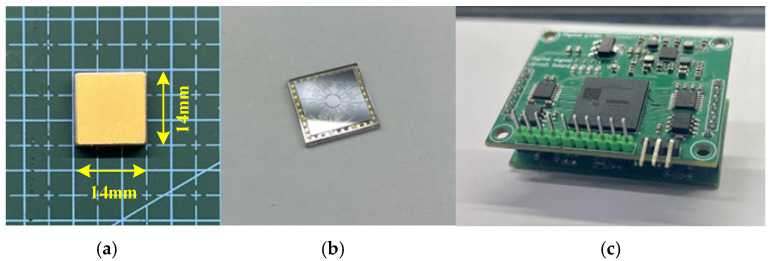
(**a**) Gyro chip package size. (**b**) Gyro physical structure. (**c**) Hardware circuit of gyro self-compensation system. Gyro self-compensation system composition.

In order to evaluate the feasibility of the proposed scale factor compensation scheme, a gyroscope self-compensation system is designed, which contains both analog and digital circuits. Figure 8a,b show the SVRG head; Figure 8c shows the analog circuit of the self-compensation system and the digital circuit of the self-compensation system. Discrete components such as the crystal oscillator, capacitor–voltage converter, and demodulation circuit together with the SVRG meter head form the analog circuit to play the role of signal amplification and conditioning. The digital system chooses an A7_XC7A100T series chip as the processor, together with high-precision ADC and DAC chips to complete the signal acquisition and processing.

**Figure 9:**
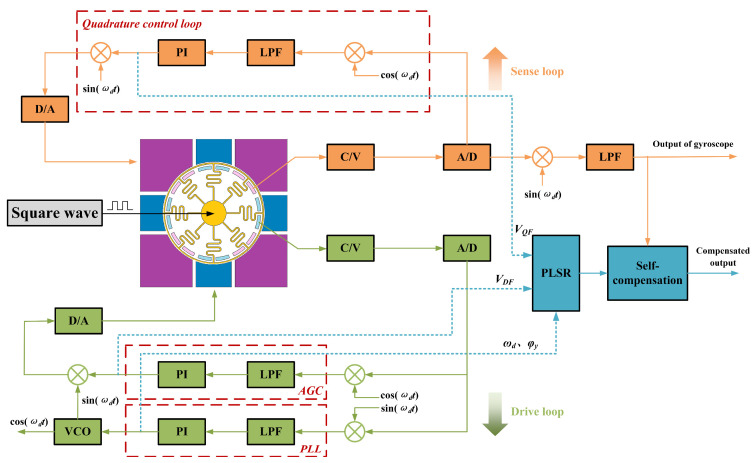
Block diagram of self-compensating system.

Figure 9 illustrates the block diagram of the signal extraction of the self-compensating system. It consists of a drive control loop and a force feedback control loop. The drive control loop achieves the frequency and amplitude stabilization of the gyroscope drive modes through a phase-locked loop (PLL) and automatic gain control (AGC). The PLL can obtain the resonant frequency information [23], and the output signal of the PI controller in the AGC is the drive control voltage. The detected modes are realized as a sensitive control loop by the force feedback control method, which accomplishes the elimination of quadrature error. The PI controller output signal in the quadrature control loop is a quadrature feedback voltage. The calibration of the demodulated phase angle can be performed in a digital system using the quadrature output signal and the detected output signal. In this way, the parameters required for the whole self-compensating system are extracted. In order to facilitate the implementation of the compensation algorithm, corresponding upper computer control software has been designed for the gyroscope control system. This software is capable of collecting and storing parameter variables in different temperature environments in the EEPROM of the digital board. Upon activation of the gyroscope self-compensation system, the stored parameter variables are first accessed in order to complete the initialization of the control parameters and the establishment of the self-compensation model.

### 5.2. Compensation Results Test

**Figure 10:**
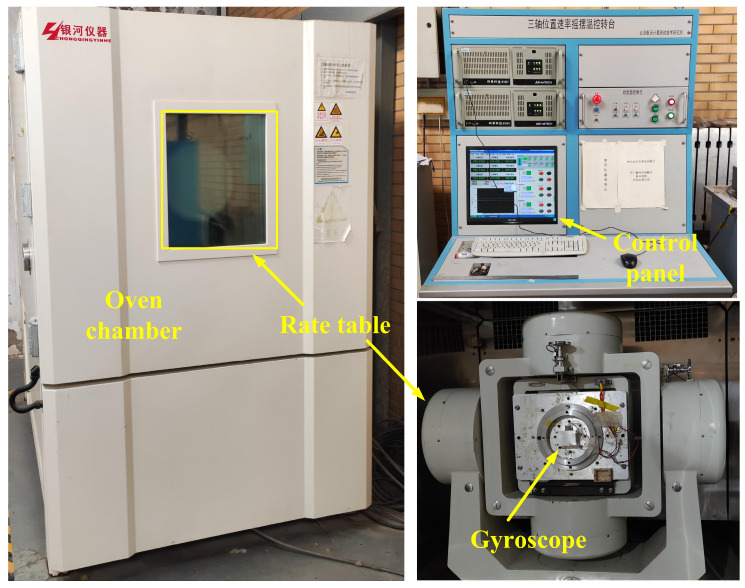
Test experiment environment setup.

**Figure 11:**
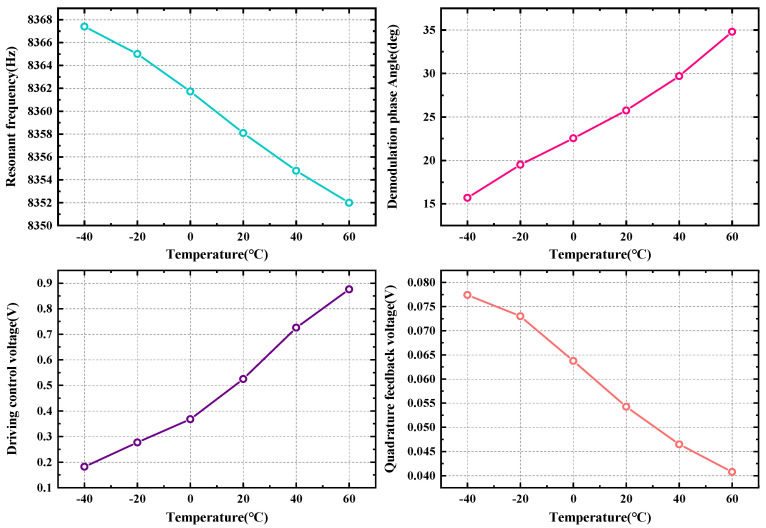
Test results of each parameter across a wide temperature range.

In order to test the relationship of the gyroscope parameters with temperature and the effect of self-compensation, it is necessary to design a complete temperature experiment. Figure 10 shows the experimental environment of the three-axis temperature-controlled rotary table. The test SVRG prototype is installed in the center of the rotary table, and the upper computer can be used to change the temperature and rotational speed and other parameters in the rotary table. We chose six temperature points from −40 °C to +60 °C to test the results. The system needs to be held for one hour at each test temperature point, and the parameter testing starts after the SVRG reaches thermal equilibrium. The four parameters of resonant positive frequency, drive control voltage, quadrature feedback voltage, and demodulation phase angle are tested first, and the test results are shown in Figure 11.

Analyzing the test results of Figure 11, the resonant frequency and the demodulated phase angle have a strong correlation with temperature and can be considered good temperature indicators. The temperature coefficient of the resonant frequency is −0.1589 Hz/°C and the temperature coefficient of the demodulation phase angle is 0.1847 deg/°C.

**Figure 12:**
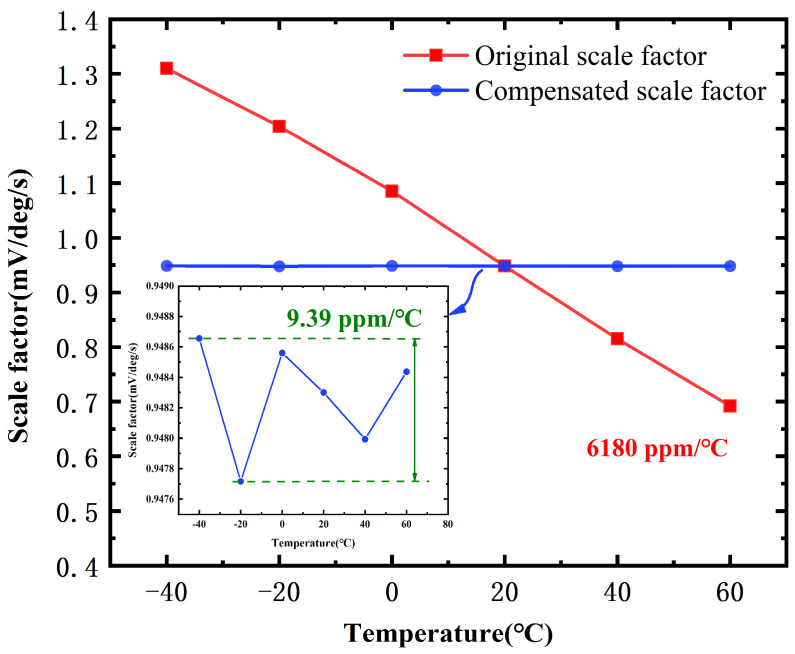
Comparison of scale factor temperature sensitivity results before and after SVRG compensation.

After starting the gyroscope’s scale factor self-compensation, revolutions per minute (RPM) tests were performed at different temperature test points. The output of the SVRG was collected at rotational speeds corresponding to 0°/s, ±1°/s, ±2°/s, ±5°/s, ±10°/s, ±20°/s, ±50°/s, ±100°/s, ±200°/s, and ±300°/s, respectively. The collected data were then processed to calculate the scale factor corresponding to the SVRG at that temperature test point. Figure 12 shows the comparison of the results before and after the SVRG scale factor compensation. The results show that the scale factor temperature sensitivity of the SVRG is 6180 ppm/°C before compensation, and the temperature sensitivity is 9.39 ppm/°C after compensation, showing a significant effect of compensation.

**Figure 13:**
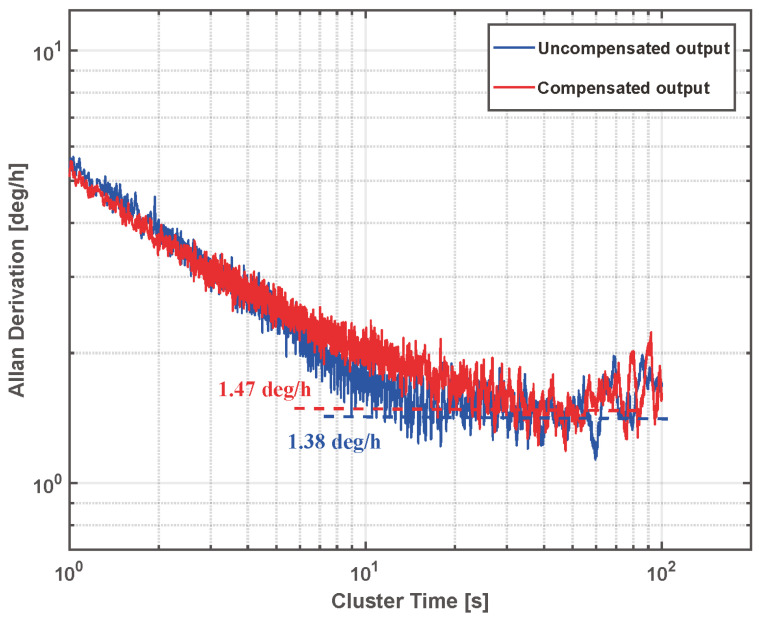
Gyroscope zero-bias stability test results before and after compensation.

The Allan bias analysis of the 1 h SVRG output data is shown in Figure 13 with and without scale factor compensation, respectively. The results show that the zero-bias stability of the SVRG before and after compensation is basically unchanged. This proves that the scale factor compensation method proposed in this paper has no impact on the static performance of the gyroscope.

Table 1 presents a comparison of previously reported work with the present study. The effectiveness of the scale factor compensation method in improving *SF* temperature sensitivity is illustrated. It can be seen that the compensation method in this paper has highly competitive results.

## 6. Conclusions

A self-compensation scheme for MEMS gyroscopes based on the PLSR algorithm with multi-parameter fusion is presented. An MEMS SVRG is analyzed as an example, given that its scale factor varies with temperature. A gyroscope self-compensation system is designed to predict the scale factor over a wide temperature range by fusing primary and secondary correlation terms affecting the scale factor through PLSR. Rather than introducing additional operations to the system, the approach uses only existing measurement information within the system for fusion, preserving system integrity. The predicted scale factor results are then used to self-compensate for the gyroscope’s *SF* temperature sensitivity.

The method is validated in a digital system. The maximum error of the predicted scale factor is 0.124% over the temperature range of −40 °C to 60 °C, and the temperature sensitivity of the scale factor is reduced from 6180 ppm/°C to 9.39 ppm/°C. This approach can also be used to achieve compensation for the bias of the gyroscope. Subsequent work will focus on the bias compensation of MEMS gyroscopes over a wide temperature range.

## Figures and Tables

**Table 1:** Comparison with previous works.

Works	Temperature Range (°C)	Temperature Sensitivity of Uncompensated SF (ppm/°C)	Temperature Sensitivity of Compensated SF (ppm/°C)	Improvement Ratio
[9]	−40–60	693	250	2.77
[10]	−40–60	-	60	-
[14]	−30–70	370	33	11.21
[24]	−30–30	717.3	5.02	142.89
[25]	−40–60	1368	282	4.85
This work	−40–60	6180	9.39	658.15

## Data Availability

The data used to support the findings of this study are available from the corresponding author upon request.
